# Biodegradable hydrogels and microbial consortia as a treatment for soil dysbiosis

**DOI:** 10.3389/fmicb.2025.1565940

**Published:** 2025-05-01

**Authors:** Renee A. Davis, Korena K. Mafune, Mari K. H. Winkler

**Affiliations:** Department of Civil and Environmental Engineering, University of Washington, Seattle, WA, United States

**Keywords:** crop health, environmental biotechnology, fungal-prokaryotic interactions, soil dysbiosis, soil microbial ecology, sustainable agriculture

## Abstract

Terrestrial microbial communities drive many soil processes and can be pushed into a state of dysbiosis upon disturbance. This dysregulation negatively impacts soil biogeochemical cycles, which threatens plant and soil health. Effective treatment of soil dysbiosis requires simultaneous restoration of multiple system components, addressing both the physical structure of soil and its microbial communities. Hydrogels with microbial consortia simultaneously remedy soil hydrodynamics while promoting microbial reestablishment. The purpose of this review is to shed light on soil management practices through the lens of soil dysbiosis. This is important to address not only for soil health and crop productivity, but also to mitigate climate change through improved soil carbon sequestration and reduced greenhouse gas emissions. This review positions hydrogels and microbes as tools for the treatment of soil dysbiosis, contributing to agricultural and climate resilience.

## 1 Introduction

Microbial organisms (e.g., bacteria, archaea, and fungi) operate in a network of multifactional communities that drive biogeochemical soil processes (Philippot et al., 2024; Sokol et al., 2022; van der Heijden et al., 2008). Anthropogenic activities can disturb the equilibrium of these communities, pushing them into a state of dysbiosis, which is characterized by an imbalance in soil microbial functionality. When soils are in a dysbiotic state, abiotic and biotic processes are negatively impacted. These altered processes push the ecosystem out of balance, hampering naturally occurring healthy plant-soil feedback loops, which directly and indirectly influence aquatic and atmospheric systems (Fra̧c et al., 2022; Giovannetti et al., 2023; Weller et al., 2002). Over the last decade, the increased use of the term “dysbiosis” in environmental studies has warranted the consideration of what defines a balanced state in agricultural settings. Both homeostasis and eubiosis are terms originating from the medical field and describe a healthy state of a person or their microbiome, respectively. They are both set relative to the definition of dysbiosis, which describes a diseased state (Iebba et al., 2016). The term homeostasis is more broad and can be used when describing the health of the ecosystem at scale (e.g., a balanced equilibrium among the kingdoms of life, such as plants, bacteria, and fungi), while eubiosis can be used when viewing soil health through the lens of microbial community composition (e.g., a balance of healthy bacteria, fungi, and archaea that promote healthy processes) (Dyke and Weaver, 2013). Therefore, when soils are in a homeostatic state their biogeochemical processes move closer to a state of equilibrium, which creates a more temporally stable system. An example of soils that have been far removed from homeostasis are ailing agricultural soils, which often have multiple issues, such as displaced microbial communities (i.e., dysbiosis), high salinity, low nitrogen (N), phosphorus (P), and potassium (K) availability, and altered pH (Giovannetti et al., 2023). The combination of these issues directly impacts the equilibrium of the ecosystem.

For example, a major reason why there has been a global decline in crop yields over the last several decades is because soils are in dysbiosis, and biogeochemical cycles are unbalanced. This decline has resulted in a critical conundrum regarding global food security and bioenergy supply (Klock et al., 2024; Wing et al., 2021), as food demand is projected to increase by 50% to support the global population in 2050 (Falcon et al., 2022). Often, management strategies prioritize increasing yields from a plant-centric above-ground perspective; for instance, by focusing solely on solutions that boost crop yield without considering other potential repercussions. However, without a shift to more holistic management approaches that focus on soil eubiosis to balance soil microbial processes, food and energy security will be increasingly harder to achieve (Amelung et al., 2020; Lal et al., 2011; Moinet et al., 2023). Therefore, we are entering a part of the Anthropocene where human impact on natural systems is so great that current management practices are not viable solutions for a sustainable future (Currie et al., 2023; Ramos and Timmis, 2021).

Due to these concerns, the focus has started to shift toward managing ecosystems through the perspective of soil dysbiosis, as it is a critical factor to be addressed in the search for climate-smart agricultural solutions (Purohit et al., 2024; Sofo et al., 2024). However, it is still not a term that is commonly used or considered amongst the broad interdisciplinary scientific community when considering the future management of ecosystems. A search for “soil” and “dysbiosis” (i.e., ((soil) AND (dysbiosis))) among peer-reviewed research articles and reviews in the JSTOR database over the last 30 years (1995–2025) returned 133 results, which narrowed to 64 articles when “agriculture” was included. However, no results were achieved when the search was refined to pair the term “soil” with “dysbiosis” (i.e., ((soil dysbiosis) AND (agriculture))), reflecting the limited attention this term has received (in some cases, the imbalance is referred to as “soil microbial dysbiosis”, but only 16 results are returned when this term was paired with “agriculture”). Therefore, the main driver of this review is to shed light on soil management practices through the framework of soil dysbiosis while emphasizing the importance of practices that promote eubiosis by strengthening soil microbial processes that can help move closer to a net state of soil and ecosystems homeostasis. Therefore, management practices that focus on addressing soil dysbiosis represent a powerful leverage point for addressing these interconnected challenges in agriculture. Certain sustainable agricultural management practices have turned to trials that apply biofertilizers with microbial consortia, many of which show promise in restoring microbial function while promoting plant health (Waltz, 2023). Biofertilizer formulations consisting of microbial consortia can address several aspects of soil geochemistry and nutrient availability, such as P and K solubilization and other plant growth promoting aspects such as phytohormone production (Zuluaga et al., 2024; Olaniyan et al., 2022; Poveda and González-Andrés, 2021). However, these products face several challenges with market adoption due to inconsistent viability, short shelf life, and unknown interactions with plants and native soil microbes (Fadiji et al., 2024; Vejan et al., 2019). Therefore, holistic solutions that aim to address soil dysbiosis via the reestablishment of eubiotic microbial communities through biofertilizer application must also consider the overall impact on the balance of surrounding systems and how practices can foster long-term resiliency.

## 2 Soil dysbiosis mechanisms and impacts

Soil microbial communities are diverse and interconnected, comprising bacteria, archaea, fungi, protozoa, and nematodes; all of which can form complex networks with plants. These complex networks exist in either balanced (eubiotic) or imbalanced (dysbiotic) states (Fra̧c et al., 2022), and this can significantly impact ecosystem services (Banerjee et al., 2018). Approximately 38% of the earth's terrestrial surface is agricultural land (Paustian et al., 2019). In these soil systems, practices such as tilling, fertilization, and pesticide use negatively impact biodiversity (Kepler et al., 2020; Wipf et al., 2021), shifting soil functional and interactive profiles, which can disrupt processes that are crucial to the balance of the system. There are many biological interactions that occur within the soil's geochemical context, where microbial activities can influence different nutrient pools, such as carbon (C) and N pools, through extrapolysaccharide secretion and necromass formation while also contributing to soil aggregation (Costa et al., 2018; Wang et al., 2021). Below, we highlight various examples of how anthropogenically induced soil dysbiosis has shifted geochemical processes out of equilibrium and the aftermath that it has on societal and planetary health.

One biogeochemical cycle that is clearly in an unbalanced state is the N cycle, and how soils are managed plays a crucial role, as soil dysbiosis causes “leaky” N systems which cause a plateau in agricultural yields. Part of this can be attributed to the fact that intensive agricultural practices disrupt key genetic functions like nitrogenase activity (which regulates biological N-fixation via bacteria), while simultaneously increasing N loading to the environment via fertilizer application. This has increased nutrient leaching, water pollution, and greenhouse gas (GHG) emissions (Daisley et al., 2022; Zhang et al., 2021; Yang et al., 2024; Wipf et al., 2021; Suman et al., 2022; Wan et al., 2025). This is of particular concern with nitrous oxide (N_2_O) emissions, which is ~300x more potent than carbon dioxide (CO_2_) on a per-molecule basis (Tian et al., 2020). Overfertilized soils are the biggest contributor to global anthropogenic N_2_O emissions (Ramzan et al., 2020). It has been shown that the application of N fertilizer can alter soil pH and N and P availability, which subsequently impacts the ammonia-oxidizing archaea (AOA) and bacterial (AOB) communities. These functional groups are vital intermediates in soil N processes (Ma et al., 2023), as AOB communities produce significantly more N_2_O than AOA (Hink et al., 2017). Further, specialized processes like N-fixation and nitrification undertaken by certain bacterial taxa are particularly vulnerable to N loading, as healthy communities that can promote closed-loop N-cycling are typically displaced under heavy fertilization (Calderón et al., 2017). This impaired biological cycling of N paired with copious amounts of N additions increases losses to aquatic systems (through leaching of nitrate) and the atmosphere (as N_2_O) (Menegat et al., 2022; Shanmugavel et al., 2023).

Agricultural practices and the induction of soil dysbiosis also negatively impairs the C cycle, which is directly linked to the N cycle (Osler and Sommerkorn, 2007). It is widely known that microbial communities play a critical role in decomposition processes, which influence soil organic matter accumulation and C-sequestration (Tao et al., 2023). Agricultural soils are typically depleted in soil C due to reduced net primary productivity, biomass export, nutrient depletion, and anthropogenic disturbances. Sustainable management practices that aim to shift an ecosystem closer to equilibrium by addressing soil dysbiosis could reverse this trend, enabling soils to act as a significant C sink with a global potential to store up to 1.85 Pg C/yr (Zomer et al., 2017). If atmospheric C could be more efficiently sequestered and soils better managed for long-term storage, climate-change mitigation efforts could be greatly advanced.

In addition to N and C cycling, water availability and P resources are strained when soils are managed in a way that promotes dysbiosis. From a water resource perspective, growing crops on a global scale is extremely water-intensive, and the recent increase in wide-spread drought has raised concern on how to increase crop yield while minimizing the use of finite water resources (Chen et al., 2016; Poudel et al., 2021). From a nutrient perspective, P is an essential nutrient for plant growth and a common fertilizer component. However, P is a nonrenewable resource derived from mined rock phosphate and is facing rising costs and geopolitical instability (Cordell et al., 2009). Therefore, how to manage ecosystems in a way that returns soil communities to balanced state is challenging because there are microorganisms that can increase soil water holding capacity through soil microaggregation (Zheng et al., 2018; Pauwels et al., 2023), others that can solubilize residual P to plant available P, and many have multi-functional benefits, such as increasing plant water uptake and resiliency, while providing plants essential nutrients.

An example of this can be viewed with arbuscular mycorrhizal fungi (AMF), which are an obligate plant mutualist and form in extensive belowground networks. AMF associate with plants, exchanging water and nutrients, such as P and N, for plant derived photosynthates (Lanfranco et al., 2018). This mutualism is ancient and broad, dating back to the terrestrial expansion of plants. Today, ~85% of land plant families associate with AMF (Strullu-Derrien et al., 2018). AMF can enhance plant productivity, as well as increase resiliency to salinity, pollution, and disease (Begum et al., 2019). Recent research has illuminated how they act as “fungal highways” and orchestrate bacterial diversity around their hyphae (Warmink et al., 2011; Zhang et al., 2022). They are also key components of the global soil C pool, storing ~13 Gt of the CO_2_ fixed by plants (Hawkins et al., 2023). To add to the complexity, plants can actively influence subterranean communities by releasing root exudates that recruit specific microbes, for example, during nutrient limitations (low N and P) or drought conditions (Williams and de Vries, 2020; Pascale et al., 2019). However, AMF abundance and network connections are disrupted by intensifying agricultural practices (particularly tilling), sending them into a dysbiotic state which can have a cascade effect on other interactive microbial communities (Wipf et al., 2021). For instance, the disruption of AMF communities can negatively impact plant nutrient allocation, soil structure, and water retention while limiting plant root exploration (Hartman and Tringe, 2019). Additionally, the impact that this has on AMF-associated microbial communities and the repercussions those impacts hold for soil biogeochemical cycling remains widely unexplored. Given these complex interactions, effective treatment of soil dysbiosis requires simultaneous restoration of multiple system components, including microbial communities, soil structure, and nutrient dynamics. Though the use of biofertilizers has started to expand with sustainability goals as a focus, there are various things that need to be considered for efficient delivery systems. For example, shifting focus away from practices that aim to only increase plant yield at the expense of soil eubiosis.

## 3 Hydrogel-based delivery systems

Hydrogel-based delivery systems hold high potential as carrying agents for biofertilizers that aim to address soil dysbiosis. Hydrogels have been studied for biological use for over 50 years (Wichterle and Lím, 1960). They can be created from natural or synthetic materials (Liu et al., 2022), but organic polymers are the most common (Patra et al., 2022; Tariq et al., 2023). The hydrogel structure consists of matrices created through either chemical methods, such as cross-linking, or physical processes, like the hydrophobic interactions among polar groups (Antunes et al., 2024). Various encapsulation technologies, including spray drying, emulsification, and ionic gelation for cross-linking are used to produce hydrogels (Vejan et al., 2019), and they can be shaped into various forms (e.g., spheres/beads). The hydrophilic matrices in a hydrogel construct can absorb and desorb water into the soil, hence regulating soil moisture (Patra et al., 2022). These structures contain micropores and allow for the encapsulation and immobilization of various agrichemicals, beneficial microbes, or a combination of both. While agrochemicals have been a primary focus, there is growing interest in co-immobilizing multiple microbial strains for synergistic effects (Antunes et al., 2024; Mafune and Winkler, 2024). Once applied, these materials degrade within 12 weeks, with degradation rates influenced by several environmental and material factors (Kurowiak et al., 2020; Wu et al., 2016).

In the context of microbial consortia, hydrogels shield microbes from environmental fluctuations and dehydration (Antunes et al., 2024; Liu et al., 2022). This protection enhances microbial survival and facilitates controlled release dynamics for nutrients, water, and microbes (Chaparro-Rodríguez et al., 2023). When applied to soil, hydrogels can induce controlled delivery of nutrients such as N, P, and K, as well as water (Hamed et al., 2024; Hu et al., 2021). In addition to prokaryotic microbes (i.e., bacteria and archaea), hydrogels can also house fungal spores, hyphae, and propagules. For instance, recent compatibility tests have demonstrated variation in AMF spore germination when co-immobilized with diazotrophic bacteria in alginate beads (Mafune et al., 2024). Combined, the versatility of hydrogels as moisture-regulators and carriers for diverse microbial consortia, chemicals, and physical components (e.g., zeolites or biochar) may offer a multi-functional competitive advantage in commercializing technology that aims to restore soils to a eubiotic state with the goal of returning ecosystem processes to equilibrium.

## 4 Restoration mechanisms

The restoration of soils in a state of dysbiosis requires the integration of practices that aim to improve soil physical, chemical, and biological properties to improve soil health/homeostasis. As this review has been emphasizing, soil dysbiosis is directly and indirectly linked to soil abiotic processes. This is why approaching the core issue of soil health through the lens of ecosystem homeostasis is important, as holistic frameworks are integral to decision making that results in restoration success (Wyant et al., 1995; Heneghan et al., 2008). By focusing in on how balanced microbial communities can promote beneficial soil aggregation, balanced pH, plant growth, C accumulation and long-term storage, and “non-leaky” nutrient cycles, scientists and engineers can hopefully be inspired to innovate restorative soil solutions through a broadened perspective.

**Figure 1:**
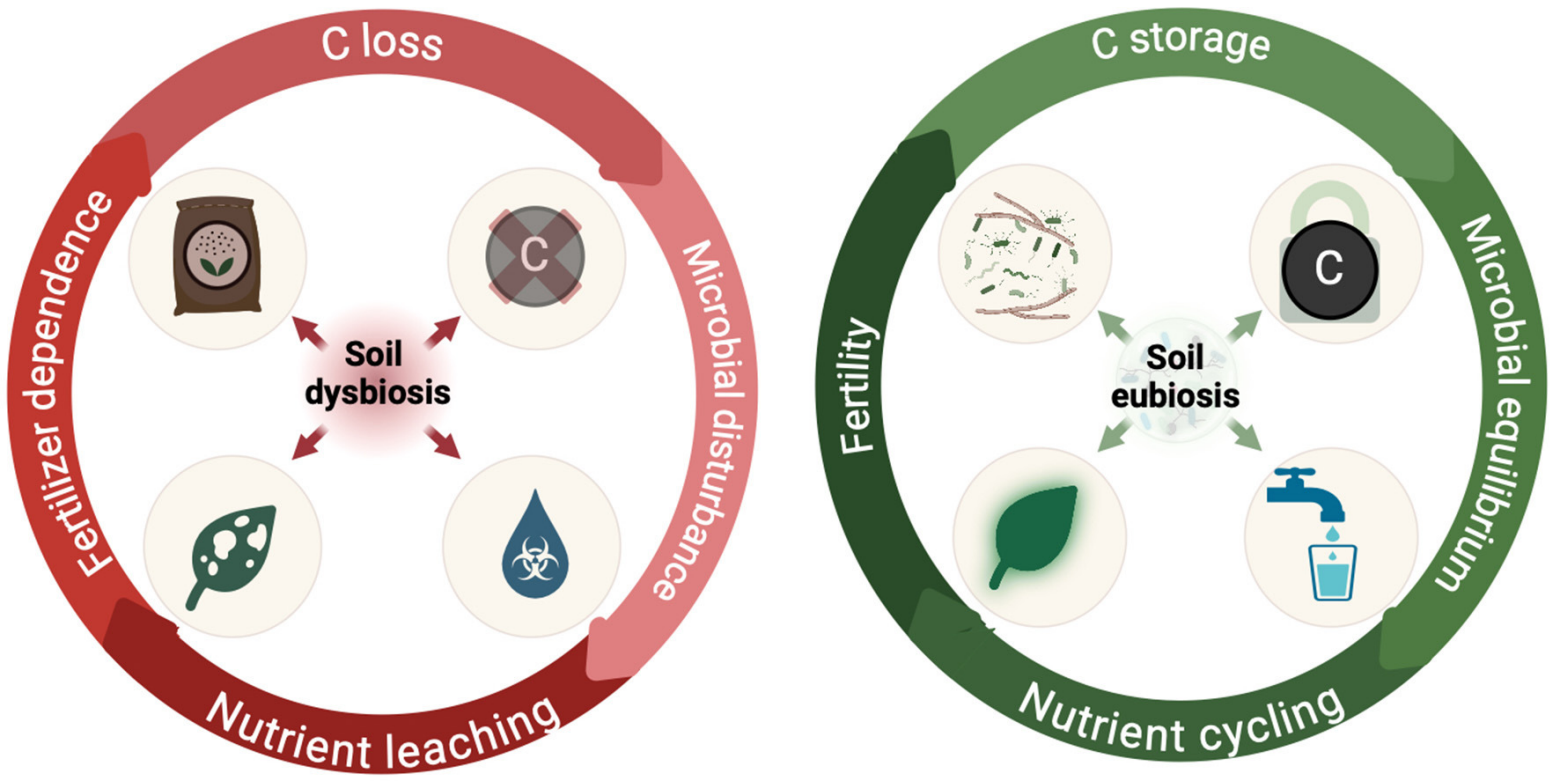
Contrasting states of soil health: dysbiosis drives nutrient leaching and depletion while eubiosis promotes nutritional increases through microbial flourishing. Inspired by concepts conveyed in Fra̧c et al. (2022), Giovannetti et al. (2023), and Purohit et al. (2024).

When considering restoration practices that can have multi-functional attributes, microbial-embedded hydrogels with a mixed consortium can simultaneously address all three areas of soil health (chemical, physical, and biological), which are all intrinsically linked to some extent in soils. For instance, in terms of soil physical properties, hydrogels undergo significant volume changes as they absorb and release water, regulating soil moisture through gradual diffusion. This leads to improved water hydrodynamics, including water holding capacity and reduced soil compaction (e.g., a decrease in soil bulk density so roots have more exploration accessibility) (Patra et al., 2022). The physical changes induced by hydrogels create conditions that support both plant root development and microbial re-establishment (Figure 1). Further, these conditions also can drive nutrient transformation and translocation, which links the hydrogels impact on soil physical properties to soil biological and chemical properties.

Another example of the hydrogel construct as a multifunctional biotechnology for soil restoration can be demonstrated from the viewpoint of soil chemistry. The hydrogel can biodegrade slow enough to support microbial activities that may drive soil organic C formation. The high-water content of the hydrogel (approximately 75% at capacity) also creates an environment that facilitates plant-microbe interactions (Aung et al., 2018; Gajić et al., 2023). Hydrogel-encased consortia can include a variety of beneficial organisms, including, but not limited to, diazotrophic (N-fixing) bacteria, phosphate solubilizing bacteria, and AMF that together drive nutrient availability and exchange. Outside of the microbial players in the hydrogel, AMF can also orchestrate a bacterial community in the rhizosphere that improves nutrient availability, e.g. through increased P mineralization (Wang et al., 2023; DiLegge et al., 2022). AMF also play an important role in soil macroaggregation, impacting soil structure, which can further support microbial growth and reestablishment (Hestrin et al., 2021). This demonstrates how a complex biological community within a hydrogel construct could benefit soil biological, physical, and chemical properties.

Further studies would be needed to shed light on how hydrogel consortia assemble, and how this assembly is influenced by soil chemical and physical properties. Hydrogels have potential to enhance synergistic community assembly by protecting microbes from environmental stressors including dehydration, pH changes, temperature fluctuations, and washout. This is particularly important for plant growth promoting bacteria (PGPB), which are often gram-negative (meaning that they have thinner cell walls) and more sensitive to environmental stressors during biofertilizer production (Chaparro-Rodríguez et al., 2023). The potential uses and applications of hydrogels with microbes and/or consortia go beyond nutrient restoration and mineralization processes. Some calcium-alginate hydrogel bead systems incorporate the entomopathogenic (insect pathogen) fungus *Metarhizium spp*. for pest control (Antunes et al., 2024). Alginate beads also have been tested in bioremediation practices, for instance, *Pseudomonas fluorescens* was encased in alginate beads to explore their efficacy on PCB degradation (Power et al., 2011).

## 5 Future directions

Biodegradable hydrogels with microbial consortia offer a sustainable solution for soil health due to their customizable nature and broad applications. However, several key knowledge gaps need to be addressed. The first priority is optimizing microbial consortia composition, and understanding how different players interact to influence soil dynamics and plant growth. Ideally, hydrogel consortia should be selected based on the ecosystem they are intended to be applied to (Mafune and Winkler, 2024), and more research is needed. PGPB serve diverse functions in soil, from N-fixation to P-solubilization and organic matter decomposition. Therefore, some species like *Azospirillium brasilense*, are versatile and often used in commercial biofertilizers (Rodriguez et al., 2004). These rhizospheric “multitaskers” are particularly valuable for inclusion in hydrogel formulations. However, repercussions of their application could be different across ecosystem types, and native communities could potentially be better suited to promote soil eubiosis. This is because interactions between bacterial species can create beneficial synergies—for instance, certain *Pseudomonas* strains can enhance the N-fixing ability of *Azospirillum brasilense*, while increased N availability in turn promotes *Pseudomonas aeruginosa* growth (Sanow et al., 2023; Marogi et al., 2024). Understanding these metabolic relationships across different soil types and in ecosystems is important for designing and engineering hydrogel consortia and synthetic communities.

An interesting pursuit in search of these solutions would be to understand how hydrogel encased communities derive C from the surrounding environment and hydrogel material for either growth or respiration, since this determines how much soil organic C can be accumulated and sequestered (Tao et al., 2023). These further impacts physical parameters such as aggregate stability and water repellency (Blanco-Canqui and Benjamin, 2013). The addition of particles to the mixed-microbial hydrogel construct (such as biochar) can also enhance how physical, chemical, and biological processes are benefitted. For example, biochars have porous surfaces that provide additional habitat for microbes while contributing to potentially long-term C sequestration (Yang et al., 2020). Spatial patterns show clustering around specific microniches, such as root and biochar micropores (Schmidt et al., 2018). Within these microniches, various levels of resource exchange and interkingdom signaling occur, highlighting the importance of a healthy rhizosphere microbiota (DiLegge et al., 2022).

To date, most research on microbial based hydrogels has been limited to greenhouse, pot, and other *in vitro* studies, though a couple of studies have applied microbial hydrogels in the field (Table 1). This demonstrates that long-term hydrogel application studies are important to evaluate real-world performance over multiple growing seasons. These studies would help determine optimal application rates and treatment durability; however, it is emphasized that major consideration should be given when selecting what microbial consortia is to be applied. This is because there is little knowledge on what repercussions are associated with applying non-native or genetically modified microbes into different soil communities (Mafune and Winkler, 2024). The degradation process of hydrogels in soils also requires further investigation. While two studies have addressed the biodegradation of alginate beads (Kurowiak et al., 2020; Song et al., 2020), to our knowledge, none have fully characterized the products of degradation, which may have further benefits in soil. For example, oligomers from alginate, chitosan, and carrageenan can support plant growth and disease protection through various pathways and mechanisms (Moenne and González, 2021). Downstream degradation products could provide additional benefits, with C-rich monomers serving as nutrients for microbes, fungi, and plants.

**Table 1:** Summary of hydrogel-based or microbial consortia agricultural studies, grouped by study type.

**Type of hydrogel**	**Inclusions**	**Release rates**	**Major findings**	**Shelf life/viability**	**Reference**
**Field studies**					
Maltodextrin and gum arabic microcapsules	*Bacillus subtilis* B99-2	n/a	Microencapsules containing *B. subtilis* were effective against *Rhizoctonia* tomato rot.	*B. subtilis* had a greater survival rate in microcapsules (87.53 ± 0.84%) than wettable powder (47.06 ± 1.49%) after 540 days in storage.	Ma et al., 2015
Sodium alginate	*Paenibacillus polymyxa* MSRH5, *Bacillus nakamurai* MSRH1, and *Bacillus pacificus* MSRH3	n/a	Microbial consortia produced indole acetic acid (IAA) and improved water retention during salinity stress in wheat plants.	n/a	Saad et al., 2020
Lignin	Ferrous sulfide nanoparticles	n/a	The composites removed 37.6% of Cd from soil and 34.5% from water spinach within 30 days, while also increasing soil nitrogen by 16.1% and organic matter by 13.8%. Soil microbial functioning was also improved.	n/a	Wei et al., 2023
Sodium alginate and chitosan microcapsules	Chlorantraniliprol	Chlorantraniliprol release increased linearly in the first 12 h, continued steadily to 48 h, and slowed from 46 to 92 h.	Microcapsules containing chlorantraniliprole were effective as biocontrols for rice stem borers.	n/a	Yang et al., 2021
Layers of alginate, chitosan, and modified starch	*Bacillus velezensis* NH-1	After 28 days in soil, the release reached 2.51 × 10^7^CFU/g. After 45 days, it increased to 10^8^ CFU/g.	Monolayer alginate microcapsules exhibited the highest efficacy (100%) in controlling *Fusarium* wilt in the field.	Viable cells detected in ALG after 65 days in dry storage.	Luo et al., 2019
**Greenhouse studies**					
n/a	*Glomus mosseae, Penicillium simplicissimum*, and *Trichoderma harzianum*	n/a	*G. mosseae* and *T. harzianum* increased plant growth more than *T. harzianum* alone.	n/a	Chandanie et al., 2009
Sodium alginate	Dinotefuran (DIN)	After 63 days, the cumulative release of DIN in hydrogels varied by soil type and temperature: Black soil: Release increased by 8.02–60.73% at 20°C and 7.70–75.23% at 30°C (compared to 10°C). Yellow soil: Release increased by 4.08–41.77% at 20°C and 6.12–46.58% at 30°C. Red soil: Release increased by 2.41–16.79% at 20°C and 2.96–31.01% at 30°C.	The release rates of DIN encapsulated in microspheres varied based on temperature and soil type, and reduced signs of copper toxicity.	n/a	Du et al., 2023
n/a	*Azotobacter chroococcum, Bacillus polymixa, Pseudomonas putida*, and *Glomus intraradices*	n/a	The consortia improved *Stevia rebaudiana* growth. Dual inoculations generally yielded better results than complex consortia.	n/a	Vafadar et al., 2014
**Pot studies**					
Calcium alginate (Ca-alg)	*Pseudomonas putida* A (ATCC 12633) and perlite	Ca-alg beads w/0.2% perlite released microbial cells at 4.2 x 10^5^ CFU/mL. Beads w/0.4% perlite released microbial cells at 8.2 x 10^5^ CFU/mL.	The beads enhanced plant growth in *Arabidopsis thaliana*, with an increase in colonization from 2.1 × 10^4^ to 9.2 × 10^5^ CFU/g soil after 21 days.	The beads remained viable for 150 days at both 4°C and 24°C, with no significant loss in viability.	Liffourrena and Lucchesi, 2018
n/a	*Funneliformis mosseae* and *Pseudomonas putida*	n/a	The consortia improved growth and defense against *Tuta absoluta*, biomass increased by 57.34% and 54.46% with single inoculations, while dual inoculation led to a 255.49% increase.	n/a	Zhao et al., 2024
Sodium alginate	*Ochrobactrum ciceri*	Slow release over the course of 30 days.	The beads were effective against against *Sclerotium rolfsii in vitro*, and improved chili plant growth.	n/a	Riaz et al., 2023
***In planta studies***					
Sodium alginate	*Pseudomonas plecoglossicida* R-67094 and *Rhizophagus irregularis* MUCL 41833	n/a	Co-entrapment of *P. plecoglossicida* improved hyphal branching of *R. irregularis* in potato plant roots.	n/a	Demortier et al., 2017
Sodium alginate	*Pseudomonas sp*. DN18, zinc oxide nanoparticles, and salicylic acid	n/a	The beads were effective against *Sclerotium rolfsii*, and improved growth in rice seedlings.	n/a	Panichikkal et al., 2021
***In vitro*** **studies**					
Sodium alginate	*Glomus mosseae* and *Trichoderma harzianum*	n/a	The percentage of viable propagules for *T. harzianum* peaked on day 6 and *G. mossea* reached its peak on day 14. Both can grow outside the alginate beads.	All treatments had similar final germination rates by day 14.	De Jaeger et al., 2011
Leather waste collagen	*Ensifer sp*.Y1	Cumulative release of N and K reached ~55% after 60 h.	Controlled release of N and K occurred over the span of 40 days.	n/a	Hu et al., 2021
Calcium alginate (Ca-alg), blended with supplemental materials	*Cellulosimicrobium cellulans* GS6	Release rates varied based on bead composition (vR/day): Ca-alg: 0 Ca-alg w/gelatin: 8.9 x 10^3^ Ca-alg w/cellulose: 13 x 10^3^ Ca-alg w/chitin: 22.8 x 10^3^ Ca-alg w/olive oil: 40.9 x 10^3^	Modifying capsule materials can improve the controlled release of microbes.	n/a	Liu et al., 2008
Sodium alginate, carboxymethyl cellulose, eggshell	Copper (Cu^2+^) ions	Slow release in a model soil solution over a period of 14 days.	The biocomposites absorbed Cu^2+^ions depending on moisture content (up to 281 mg g−1 for wet and 49 mg g−1 for dry composites).	n/a	Skrzypczak et al., 2019
Calcium alginate	*Saccharomyces cerevisiae* and *Beauveria bassiana*	Variable.	Microbial consortia affected CO_2_ release rates, an attractant for agricultural pests including western corn rootworm larvae.	n/a	Vemmer et al., 2016
Sodium alginate and chitosan microcapsules	*Trichoderma viride*and Cu^2+^ ions	*T. viride* spore release lagged initially, then rose exponentially. Cu^2+^ ions had a faster initial release, which eventually slowed over a period of 14 days.	Electrostatic interactions between microscapsule materials affected encapsulation efficiency and stability.	The presence of Cu^2+^ ions did not affect *T. viride* spore viability.	Vinceković et al., 2016
Sodium alginate	Glyphosate (Gly) with attapulgite	60% of Gly was released in alkaline solution (pH = 8.5) within 70 h (the rate was 20% in neutral and acidic pH conditions).	pH impacted release rates of Gly. Alginate offered better protection against UV and temperature fluctuations, retaining 95% of Gly after 300 h of exposure.	The half-life of Gly in soil was 65.89 h, and Gly encased in hydrogel had a similar half-life (66.27) whereas attapulgite-Gly had a shorter half-life of 31.18 h.	Zha et al., 2022

From a commercial perspective, several factors will influence market adoption of biodegradable hydrogels with microbial consortia. The plant growth-promoting properties of microbial-encased hydrogels suggest they be used as a biofertilizer, either as supplement or replacement for conventional fertilizers. However, successful commercialization will depend on achieving cost-effective manufacturing at scale, while concurrently balancing chemical inputs to soils. The cost of fertilizers has been rising and when paired with the concern of widespread soil dysbiosis, farmers face challenges sustaining profitable yields. For example, ammonia production has been steadily increasing over the last few decades, and costs fluctuate between 400 and 1600 USD/t because of transportation costs from centralized manufacturing centers, as well as natural gas prices and sources (Fernández et al., 2024; Zhang et al., 2020). Additionally, the production of conventional N fertilizers is energy-intensive and accounts for approximately 2% of global CO_2_ emissions (Menegat et al., 2022). P is a non-renewable resource and comes with its own set of rising costs and geopolitical instabilities (Brownlie et al., 2023). This combination not only puts stakeholder livelihoods at risk, but it also contributes to the looming concern of global food security. Therefore, there is a need to determine the energy, production, and application costs of hydrogels at scale. There is also a need to understand the potential for this biotechnology in the Carbon Dioxide Removal (CDR) market, while also including emissions other than CO_2_, such as N_2_O. As N_2_O is ~300x more potent than CO_2_, is emitted largely from fertilized soils, and if the reduction is prioritized, it could greatly benefit the environment, as well as boost CDR market efforts and reach (Tian et al., 2020).

As high-throughput culturing technology advances, there may be a viable niche for cost-effective and labor-efficient customization of enrichment, isolation, and reapplication of site-specific, well-adapted microbial consortia. These would have the added benefit of being non-invasive and naturally evolved to dominate and adapt to local conditions. It could provide an avenue to introduce high-performing strains into the field at higher concentrations, further accelerating N and C translocation and boosting plant yield.

Overall, *in vitro* and greenhouse studies have provided a foundation for understanding microbial consortia encased in a hydrogel construct. Moving forward, research into microbial synergies, field application methods, ecological impacts, and biodegradation profiles are key. Efforts along these lines will not only advance the scientific understanding of hydrogel-microbe interactions, but also provide the evidence needed to accelerate adoption of this promising biotechnology for sustainable agriculture and restoration practices beyond the agricultural sector.
